# Life under lockdown and social restrictions - the experiences of people living with dementia and their carers during the COVID-19 pandemic in England

**DOI:** 10.1186/s12877-021-02257-z

**Published:** 2021-05-10

**Authors:** Remco Tuijt, Rachael Frost, Jane Wilcock, Louise Robinson, Jill Manthorpe, Greta Rait, Kate Walters

**Affiliations:** 1 Research Department of Primary Care and Population Health, University College London, Royal Free Campus, Rowland Hill Street, London, NW3 2PF UK; 2 Newcastle University, Newcastle upon Tyne, UK; 3 King’s College London, London, UK

**Keywords:** Dementia, COVID-19, Social restrictions, Social engagement, Carers, Qualitative Research

## Abstract

**Background:**

The impact of COVID-19 restrictions on people living with dementia and their carers is an emerging focus of recent research determining how we can best support this population. People living with dementia have faced service curtailment, increased risk for COVID-19, as well as potential heightened deterioration. This study reports the experiences of people living with dementia and their family carers during the early months of the COVID-19 pandemic in England and the impact on them.

**Methods:**

We recruited and remotely interviewed 30 people living with dementia in their own homes and 31 family carers, via video or telephone call in mid-2020. Data were transcribed and analysed using thematic analysis.

**Results:**

People living with dementia often had a basic understanding of COVID-19 restrictions but could have difficulty translating this into personalised risk-appraisal of their own actions. Managing COVID-19 risks facing people living with dementia at home was largely done by family carers, exemplified by changes to living arrangements, which could strain or sustain caring relationships. Well-established familial caring relationships contributed to the wellbeing of the person living with dementia and their carer, as well as keeping to simple routines that included leaving the home for exercise and stimulation. People living with dementia reported some negative psychological and cognitive effects due to the imposed restrictions, such as increased apathy, irritability, or anxiety, which were fuelled by lack of social engagement.

**Conclusions:**

Structuring routine (remote) social interactions where possible could increase social engagement and improve wellbeing for people living with dementia, especially those with limited familial support in a post-COVID-19 context. As some care relationships had been restructured to manage COVID-19 risks, additional carer strain may emerge as a result of the impact on the independence of the person living with dementia and come to the attention of professionals in health and care services. People living with dementia and their carers highlighted the importance of maintaining or adapting routines which may be useful learning for professionals, although additional support may be necessary for those who are impacted by more severe or worsening symptoms of dementia.

**Supplementary Information:**

The online version contains supplementary material available at 10.1186/s12877-021-02257-z.

## Introduction

There are an estimated 50 million people living with dementia globally, and this is expected to increase to over 152 million by 2050 [1]. As a degenerative neurocognitive syndrome, dementia can affect cognition, speech, and judgement resulting in difficulty in maintaining general everyday activities [2]. Most people are supported by family members across the trajectory of the dementia syndrome. Supporting someone with dementia can include provision of emotional support, assistance with activities of daily living and provision of medication or personal care, underpinned by relationships and commitment that seek to maintain the values and personhood of the person living with dementia [3, 4].

With the onset of the severe acute respiratory syndrome coronavirus 2 (SARS-CoV-2) in early 2020, government restrictions were enacted to contain the virus spread. The United Kingdom (UK) government imposed a nationwide lockdown from 23 March 2020, which lasted over 12 weeks. People at high risk were instructed to ‘shield’, and refrain from contact outside their homes, while other people over the age of 70 were deemed at moderate risk and instructed to practice strict social distancing (‘self-isolate’). Although most people living with dementia were included in the age-related risk group, dementia itself was not listed as an increased risk factor for COVID-19 at the time [5]. Subsequent findings that having a diagnosis of dementia is the largest independent risk factor for hospitalisation due to COVID-19 for adults over the age of 65 [6], should encourage a more disease targeted approach to protecting those at increased risk in any future outbreaks.

Commentators have highlighted the potential deleterious impact the restrictions may have on people living with dementia, listing the disruption to routines, increased isolation, lack of respite, closure of day centres, loss of home or domiciliary care, and the potential of accelerated cognitive deterioration [7–10]. There is initial quantitative evidence to suggest that lockdown restrictions have resulted in increased neuropsychiatric symptoms such as depression and anxiety in a minority of people living with dementia [11–15], although there are also reports that many people living with dementia were managing optimally [16]. Other reports have highlighted the negative impact on family carers [17, 18], with initial qualitative findings from India showing increased difficulty in caring as a result of the decrease in social support and care services due to COVID-19 restrictions [19]. Other qualitative work conducted in the UK during the pandemic has shown the difficulty of decision making for carers regarding whether to permit domiciliary or home care visits during the pandemic [20] and access to other social care services [21], as well as initial exploration of the impact on the well-being of people living with dementia and their carers in Canada [22].

The aim of this study is to explore the experiences of lockdown and social restrictions on people living with dementia from the perspectives of people living with dementia and their family carers. It is important to highlight the voices of people living with dementia [23] about their experiences during COVID-19 if services are to support them and their carers, and reduce the need for crisis care or unwanted moves from home [24]. This will additionally provide guidance for future measures to support people living with dementia in the community in similar crises to the COVID-19 pandemic. This present study reports the experiences of the impact of COVID-19 restrictions in England from the perspectives of a range of people living with dementia at home and their carers.

## Methods

### Design and setting

This paper reports findings from the first wave of a longitudinal qualitative study that aimed to explore the experiences of people living with dementia and their carers in the community regarding their dementia related care. When the impact of COVID-19 and relevant restrictions began to emerge, amendments were made to the study design and protocol to conduct remote interviews and include COVID-19 related questions (data regarding remote healthcare consultations during COVID-19 are reported elsewhere [25]).

### Recruitment procedures

Recruitment used purposive sampling, aiming for a range of participants with varied type of dementia, gender, age, ethnicity, and rurality. Participants were recruited from diverse areas including memory services in East London as well as primary care practices in North London and Thames Valley; this was supplemented by online national recruitment through Join Dementia Research [26] and the Alzheimer’s Society Research Network [27]. The study received ethical approval from the London South East Ethics Committee (20/LO/0006).

Participants were screened by practice staff for eligibility using the following inclusion criteria:

1. diagnosis of dementia and living in the community
2. capacity to take part in an interview
3. able to complete an interview in English.

People living with dementia under the age of 65 were excluded as this group has been shown to incur different needs and challenges that are often specific to young onset dementia [28], and a separate research study was ongoing with a specific focus on young onset dementia [29] . Eligible individuals were sent an invitation letter and a study information sheet by post or by email, after which they could contact the study team to express interest. A researcher then provided further information about interview procedures and checked the understanding of the potential participant. Before scheduling the interview, participants were asked to identify a suitable carer the study team could also approach for participation in the study, as well as their preference for how they would like the interview to be conducted (video or telephone call). There were no further inclusion or exclusion criteria applied to carers. In this paper, as in UK policy and legislation, the term ‘carer’ refers to a family member or friend that the person living with dementia identified as someone involved in their care and who supports them. Initial interviews were offered individually, but if the person living with dementia and their carer requested to be interviewed at the same time, a dyadic interview was conducted. Those interviewed together were offered the chance to speak individually following the interview to provide further information or follow up any points raised during the interview.

### Data collection

Informed consent procedures were audio recorded with permission with careful explanations provided to each participant, point by point. Interviews were semi-structured using prompts and followed a topic guide that had been developed for this study by the authors who were experienced qualitative researchers (see Additional File 1). The topic guide was then discussed with a person living with dementia and a former carer in its initial and final versions. Interviews were conducted by RT, a male PhD candidate with experience and interest in conducting qualitative research with people living with dementia, using the remote method preferred by the participants in their own home (this was via telephone for all but one dyadic interview that was conducted via video call). RT made field notes following each interview detailing the main topics discussed, including reflection on what the participants specifically highlighted. Demographic information including age, ethnicity, type of dementia, residential status and relationship to each other, was collected at the interview’s end.

**Fig. 1:**
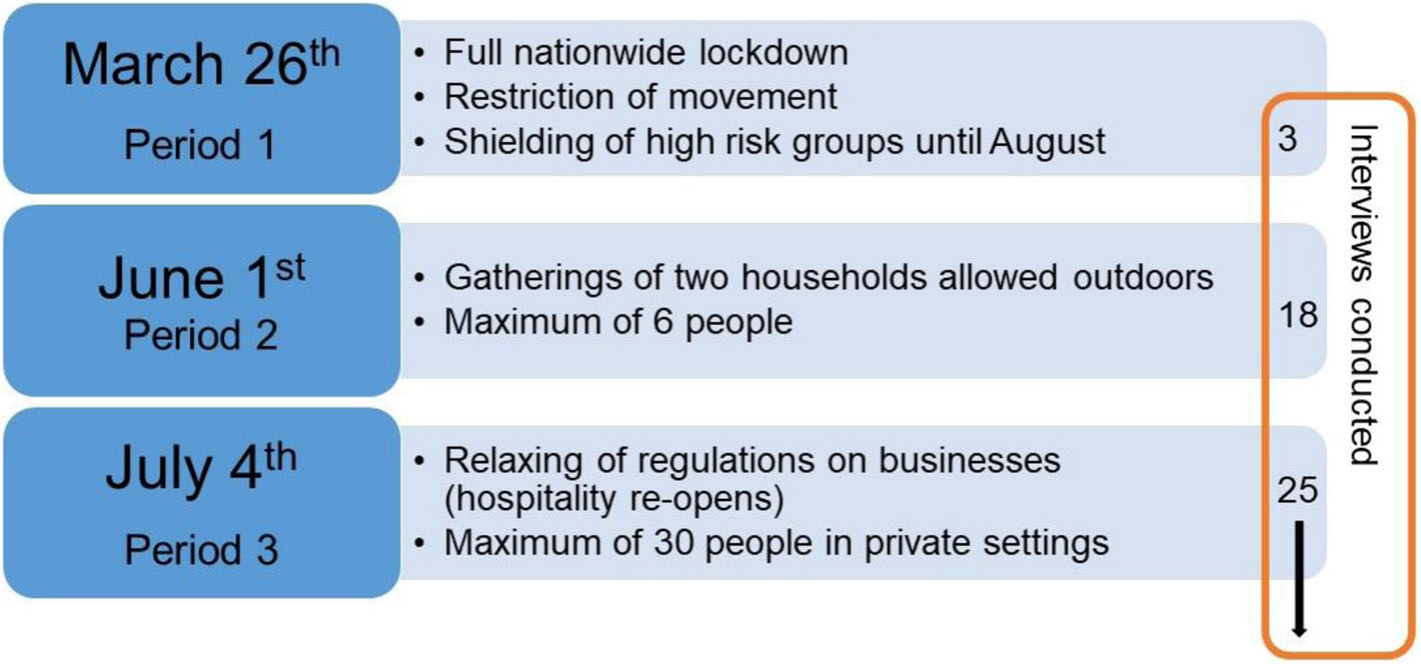
Relevant UK restrictions and time periods when interviews were conducted

Interviews were conducted between May and August 2020, when varying restrictions and social distancing requirements were in place across the UK. A summary of the different ‘periods’ is depicted in Fig. 1, and includes the number of interviews conducted in each period.

All interviews were audio-recorded and sent for transcription by a third-party company. Six randomly selected audio files were transcribed by the first author for data immersion. The first author checked all transcripts against the original audio file for any discrepancies while also pseudonymising the final transcripts. To facilitate analysis transcripts were then imported into NVivo software (version 12) [30].

### Data analysis

Reflexive thematic analysis was used to analyse the data, coding line by line and then grouping themes together where codes described similar experiences [31]. Individual interviews and dyadic interviews were analysed in the same manner. References to other participants within interviews were clarified using their relevant participant code. The authors used an inductive approach to generating themes, with two authors (RT & RF) initially coding the COVID-19 data from the first 20 interviews and reviewing field notes made after the interviews. Following their initial discussion, themes were refined and synthesised as appropriate, using a data-driven approach. Analysis of the further interviews was undertaken by the first author, which developed new themes or expanded previously established ones. The overall interpretation, meaning and saturation of each theme were discussed in several team meetings, so each author could contribute insight from their area of expertise, including dementia, caring, ageing, and qualitative research. Due to time considerations transcripts were not returned to participants for checking.

### Participants

A total of 43 people with dementia were approached by the research team following an expression of interest (20 from the Memory Services, 15 from online recruitment and eight from primary care practices). Twelve individuals declined to take part after being provided with further information due to time and effort considerations, mostly from the online recruitment strategies. Interested people with dementia were asked to identify a carer who could also participate in an interview, as well as to discuss the study information with them if they had not done so already.

A total of 46 interviews, split between 31 individual interviews (average time: 22 min) and 15 dyadic interviews (average time: 39 min), were conducted with 30 people living with dementia and 31 carers. One person living with dementia declined to take part once the interview had been arranged but their carer was interviewed. This sample comprised a heterogeneous group with varied ages (range 72–100 years), carer relationships (spouse, adult child or friend), a variety of ethnic groups (white British *n* = 37; other ethnicities *n* = 24) and different dementia diagnoses (see Table 1).

**Table 1:** Demographics of people living with dementia (*n* = 30) and their carers (*n* = 31)

	People living with dementia		Carers
Gender (n)			
Female	17		20
Male	13		11
Ethnicity (n)			
White British	19		18
South Asian	4		5
Black Caribbean	4		4
White Other	3		4
Dementia type (n)			
Alzheimer’s disease	9		
Unspecified	9		
Mixed	7		
Vascular	5		
Living status (n)		Relationship (n)	
With carer	20	Child	15
With other family	4	Spouse	14
Sheltered accommodation^a^	4	Friend	2
Alone	3		
Age range	68–100		45–84
		Spouses (mean)	76
		Children (mean)	57
Diagnosis year (median)	2019		
Range	2011–2019		

^a^No care staff on site, often includes adaptations for disability

#### Findings

Five main themes were derived relating to: 1) awareness of restrictions, 2) restructuring caring relationships to manage COVID-19 risk, 3) protective factors, 4) the psychological and cognitive impact of restrictions, and 5) the importance of social engagement.

##### Awareness of restrictions

People living with dementia discussed the restrictions imposed due to COVID-19, such as the lockdown or social distancing, and most showed some awareness and understanding of them in general terms. Acceptance of the situation was discussed positively by some, although more often reflected a negative impact of being restricted to the home or not being able to see others. The extent of the social distance restrictions that came into effect following the March 2020 lockdown, and their impact were discussed by people living with dementia as confusing:

I’ll go, ‘oh yes, I’ll go and visit so and so at this place’, and so on, ‘oh dear, *can* I just visit them? *Can* I go to this place or that place?’, you know. But, I find it hard. I find it difficult… not difficult but you know, I find it annoying more than anything, that suddenly you have to say, ‘*oh dear’*. – Person living with dementia 3001 (Male, 83)

Some carers highlighted how people living with dementia struggled to understand the restrictions and their reasons for them, notably during the height of the lockdown when people were only allowed outside their home for a limited amount of time:

He wants to go out every day, he has no grasp on this lockdown. Every day he says ‘why can’t we go here’ or ‘why can’t we go there’, and I say for the hundredth time, ‘because’, you know, ‘we’re in lockdown’. – Carer 0202 (Wife, 73)

These carers felt frustration at having to continuously respond to the person living with dementia by explaining the lockdown restrictions or reminding them about the current pandemic. Whilst people living with dementia felt they understood the restrictions, some carers noticed that even if there was some understanding or awareness, it seemed limited:

Well, yes, I do feel all right about [social distancing], because I understand why she’s [daughter] being like that, so that’s it, it’s as it should be, because that’s what we were told, she’s doing what she’s told to do and so I go along with what she says. Yes, you can’t do really more than that, can you. – Person living with dementia 1501 (Female, 89)

You think that she understands the virus and what’s going on, but then you may say something and it makes you realise, oh, she’s really not… So, you have to continually say it to her, why something’s happening, she won't be, oh yes, that’s because of the virus. It’s, oh, why’s that happening? Because of the virus. So, she’s not completely aware of it. – Carer 1502 (Daughter, 66)

Despite the potential of frustration, these carers emphasised it was important to discuss the restrictions and their implications, whether these had a direct impact on activities the person living with dementia wanted to engage in, or the effect they had on other family members or friends.

##### Restructuring caring relationships to manage COVID-19 risk

People living with dementia and their carers both acknowledged the risks regarding COVID-19 and the subsequent adjustments many made to address this. People living with dementia gave examples of not having anyone visiting their home, including other family members:

I never let anybody come into our home. I realised it was very important. [..] I was a bit mad about it, almost. – Person living with dementia 2801 (Female, 81)

In some instances, this included pausing private care workers or delaying seeking support for other home care. However, the participants in our sample that were reliant on services for personal care (e.g. help with bathing, food preparation) continued these arrangements.

Participants living with dementia in this study had been instructed to self-isolate due to their age (almost all were aged over 70), which also included most spousal carers. They usually mentioned decisions related to COVID-19 as a risk for both, but if there were particular implications for the carer these were sometimes highlighted and presented further barriers to doing everyday activities:

We haven’t been able to do that [shopping] because I was extra vulnerable, I couldn’t even go out for a walk because I was too high a risk, and I have a breathing problem. – Carer 1402 (Wife, 84)

There seemed to be substantial differences between the experience of spousal carers and adult child carers based on their co-residence. As many of the adult children in this study were not co-resident, changes in how they provided support became necessary almost immediately. For some personal contact sharply declined:

I see her at least twice a week, it used to be more, but obviously with the virus, we go in only when really necessary, now. - Carer 1502 (Daughter, 66)

Well, I mean, at the moment, it's not normal circumstances. And during lockdown it was only when permitted for garden visits. And I mean at the moment, it's still really only garden visits. So once a week. - Carer 2002 (Daughter, 52)

In some cases, for people living with dementia supported by family members who did not live with them, support from multiple extended family members had been reduced to support from just a single individual. While this could, at times, increase their sense of isolation or loneliness, discussed more in-depth in themes below, people living with dementia showed appreciation for the continued support they were able to receive from their carer:

So, what we decided was that instead of all of us visiting, only I would visit, because we just wanted to reduce any chance of him catching the coronavirus. - Carer 1202 (Daughter, 51)

Some of those supporting a parent had made the decision to either move in temporarily or move their parent in with them (in our sample these were all White British). This was not exclusively for people living with dementia who did not have a spouse, as there were also examples of children moving back home to help support both parents. A large consideration was the impact of dementia on their relative and if this would make it difficult for them to understand and adhere to the restrictions, and calculating that co-residence would make it easier to keep them safe:

There’s no way mum would be all right on her own, not in that apartment, because she would just go out and about chatting and hugging and taking the dog out for the walk and going to the shops. So, there’s no way really, being able to monitor or moderate that behaviour without having her here. – Carer 1702 (Son, 49)

Such decisions were not made lightly, since the move disrupted life for the person living with dementia and in some cases put strain on the relationship between the carer and their relative. For people living with dementia, this impacted on their independence and autonomy, which was already limited following these changes in living situations. For carers who moved in temporarily, this could also complicate life:

I’m finding it quite hard, because I get in such a muddle because I’m half living in here and half at home. – Carer 1102 (Daughter, 64)

For those adult child carers who had taken on this prime carer role, stresses mainly arose when there was a need for continual supervision which made managing daily tasks much more difficult. These difficulties often remained when lockdown restrictions eased (see Fig. 1), and there was the opportunity for some respite:

I was in a bit of meltdown last week, because everything else regarding lockdown was opening up, but nothing was opening up for me, because I can't leave Mum. It’s a little bit better now because I get the odd day when my sister has her. So that’s helpful. – Carer 0902 (Daughter, 65)

Overall, especially as restrictions eased, there was a sense that newly permitted limited engagement with other people needed to be balanced with managing the risk of contracting COVID-19. This most commonly took the form of decision making about other members being reintroduced into the household.

##### Protective factors

Participants’ perceptions of how well they were managing varied, but there were examples of people living with dementia who reported they were doing well. People living with dementia often credited this to the support of their family, with whom they had a well-established and beneficial caring relationship:

I don’t feel so confused as I did before probably because I’m with my family, I’m sure. – Person living with dementia 1701 (Female, 78)

Practical support from family members provided a sense of security and was said to sustain the well-being of both the person living with dementia and the carer. This was from both those who had moved in to support the person living with dementia or those who found other ways to help with tasks:

Absolutely fine, really. No problems whatsoever. We do put together our shopping list, whatever we need. The children will go and get whatever we need. We are looked after by our family. – Person living with dementia 1801 (Female, 72)

Some carers noted that the reduction in activity following the restrictions had helped refine a more limited routine. People living with dementia who were able to continue regular daily activities at home seemed able to manage well, maintaining activities such as gardening, doing crosswords, or reading. If the activities done at home were not very complex, this provided the person living with dementia an easier way to manage their daily routines:

He’s just really surprised us all. It seems like, it almost feels like the taking down to the very simple routine that he set himself in the home and not going out very much, seems to have helped him manage. – Carer 1202 (Daughter, 51)

Participants gave few examples of specific things that helped them to stay well, although one person living with dementia highlighted that being financially secure benefited their wellbeing:

Int: What do you think has been helpful to be able to manage quite well during the coronavirus recently?

Ppt: Truthfully? That I’ve a few bob [money] and can afford to do things. – Person living with dementia 2701 (Male, 75)

For both the carer and the person living with dementia, being able to get out of the house seemed an indicator of whether they were doing well. This typically meant either being outside in a garden, or going for a walk to a park, often described as part of a newly established routine for upkeep of physical exercise in a socially distanced manner:

No, she hasn’t been depressed or anything like that [..]. when we started going out walking in the park, it wasn’t so bad, we’re getting out, and she’s doing all right. – Carer 2502 (Husband, 76)

##### Psychological and cognitive impact of restrictions

People living with dementia who previously had been more independent and able to go outside the home found it difficult to adjust to the disruption to their usual activities. More notable and distressing impacts on mental wellbeing, mainly in terms of anxiety and irritability, were reported by people living with dementia who were aware of a decline in their independence:

There are times in the day where I get very irritable, why, I don’t know, perhaps it’s anger at myself that I can't do what I used to do, and it is very depressing at times. - Person living with dementia 0801 (Male, 73)

I feel as though I'm much more wobbly than I was, and I’m frightened, much more frightened than I was. – Person living with dementia 1601 (Female, 68)

This anxiety also related to the uncertainty about the present and the future, specifically over COVID-19 restrictions and if life would return to normal:

We’re all sort of at sixes and sevens, if you know what I mean. Nothing is as it always was. So, hopefully things will improve, but we don’t know which way it’s going at the minute. – Person living with dementia 2001 (Female, 82)

Some carers also noted an increase in irritability and apathy among their relatives:

In my view I think he’s actually declined a bit as a result of the lockdown restrictions. And to me that only shows how good it was for you to actually go out by yourself and to hang out with your cronies at the mosque. It was of tangible value. You talk about missing it absolutely there’s that aspect. But I can see the change in you. [You’ve] just become more withdrawn. – Carer 2102 (Daughter, 45)

The memory itself and also he's getting more irritable and our interests, just general things as the day goes by that you notice are different from what they used to be. – Carer 1402 (Wife, 84)

With regards to memory deterioration, some carers felt this was happening but ascribed this to the regular progressive nature of dementia. In a similar vein, some carers considered that it was perhaps their increased proximity and heightened awareness of their relative living with dementia that were leading to their assessment that their relatives’ dementia was getting worse:

I think it’s probably getting a little bit worse. [..] I don’t think the lockdown’s got anything to do with it, I just think it’s his memory is getting worse as the time goes on. But not necessarily because of the lockdown. It’s a progressive thing. – Carer 2402 (Wife, 71)

Maybe I’ve just noticed it more because I’ve been here with her. [..] Maybe this just happened anyway, and I just wasn’t aware of it. – Carer 1702 (Son, 49)

In contrast, people living with dementia who were experiencing more severe memory problems ascribed this to lockdown. For them, whether they would return to their previous capabilities was a worrying uncertainty:

Until quite recently I’ve- have been independent, and this lockdown thing has absolutely buggered my mind, excuse my French. Oh, dear, that’s very rude, but it’s just been, I don’t know if I’ll ever quite recover from it, because I’ve been in such confusion. – Person living with dementia 1601 (Female, 68)

##### Importance of social engagement

Among the changes to routines that people living with dementia and their carers reported, most found it difficult to replace those regular activities which involved a social element:

We have been going to the memory things we do, but of course that’s been curtailed at the moment. We used to go to an exercise group once a week. And then there’s a memory café every other month. [..] But of course, that’s not happening at the moment which is a bit of a shame. So that’s that. – Carer 1002 (Wife, 73)

They [used to have] a little social gathering. And I think that’s what Mum’s missed during this lockdown. – Carer 0902 (Daughter, 65)

For people living with dementia with a more recent diagnosis, there was no chance to engage with services that would have otherwise provided this routine of social interaction. The benefit of services like day centres or other community social groups was emphasised with similar sentiments:

In an ideal world, it would be great if [person living with dementia] could perhaps go to a day centre and then she’d have social interaction with other people and people to speak to [..] but obviously that hasn’t happened because of the lockdown. – Carer 2602 (Son, 56)

Carers reported that alternative activities were being arranged, using online technology such as Zoom or WhatsApp. However, this was not always a satisfactory replacement for regular social activity. Some people living with dementia struggled with the necessary technology, and even telephone calls did not always replace the social engagement that benefitted people living with dementia:

The disruption in her routine, not being able to see friends, even though I know that they have rung her, but she doesn’t always remember that. She will see me on Monday but have forgotten about that by Wednesday. – Carer 1602 (Neighbour, female, 67)

Restrictions on social engagement were viewed as one of the main detrimental effects that people living with dementia experienced during the first UK lockdown of COVID-19. In some cases, this was as impactful as changes to routines and independence mentioned in previous themes; this was especially apparent for the three participants with dementia who lived alone:

I feel, since the lockdown, I feel very bad. When I go to the mosque there’s plenty of people to talk to. Now only my family, my grandchildren, wife and children you see. The same people. – Person living with dementia 2101 (Male, 81)

It’s been dire, really awful, [..] particularly because I couldn’t see my friends. Because I’m a single person, it’s absolutely… It’s kind of awful, it’s just been awful. Horrible, really horrible experience, it’s been. And today is the first day that I’ve spoken to anybody about it. – Person living with dementia 1601 (Female, 68)

After the strict lockdown restrictions eased slightly in June 2020 (see Fig. 1), there was more opportunity to socialise. However, if people living with dementia were experiencing apathy they did not engage socially as much as they had previously:

I must admit I was surprised, because once the kids (children) could come in the garden, I thought she’d be really keen to see them and she’s not. And that is the strangest thing to me, because she was always so pleased to see the kids. - Carer 1102 (Daughter, 64)

## Discussion

People living with dementia and their family carers discussed the impact of COVID-19 restrictions, highlighting difficulties with understanding the implications of restrictions, changes to living arrangements, increased cognitive and psychological difficulties, and a lack of social engagement. Keeping to routines, in addition to carer support, provided people living with dementia with consistency that seemed to help coping with COVID-19 restrictions. Those living with dementia showed general awareness of the COVID-19 restrictions, but could experience difficulty in assessing risk for themselves or others. Carers reported needing to constantly remind the person living with dementia of the current situation and specific limitations currently affecting them. While this might lead to frustration, it seemed important for those engaging with people living with dementia to provide clear explanations that are not just reminders but also highlight the implications of restrictions and decisions related to this [32].

The psychological impact of COVID-19 restrictions, as well as the impact on the symptoms of dementia, was notably more evident in those who, before the onset of COVID-19 restrictions, had more routine social activity outside the home. Missing social engagement led to increased feelings of isolation, particularly for participants who relied on these social routines or lived alone. Social engagement for people living with dementia has previously been described as important to retain the sense of self [33], which is in line with the growing literature exploring a social health model of dementia [34]. In the general older population, similar to the findings in this study, it was reported that increased loneliness alongside reduced physical activity were risk factors for worsening mental health during the COVID-19 pandemic [35]. People living with dementia who could create new or adapt old routines (social or otherwise) to their home environment appeared to cope better. This included being able to get out for physical exercise or a break.

Management of COVID-19 risk was undertaken primarily by family carers, and this put pressure on caring relationships due to the resulting negative impact on the autonomy of the person living with dementia, and the disruption and changes to carers’ lives. Although there were examples of positive experiences, our sample largely reported negative experiences. The difficulty of supporting the independence of a person living with dementia whilst managing risk has previously encouraged ‘acceptable’ risk-taking as part of person-centred care [36, 37]. Ensuring awareness and understanding of restrictions as well as their implications are essential in supporting person-centred care during hazardous risk management decisions, like those potentially encountered during the COVID-19 pandemic. This may include decisions around (dis)continuing paid home care, which was discussed by only a few participants in our sample but has been the focus of other recent qualitative research [20].

Changes to carer relationships were often made to provide additional support to the person living with dementia, and this support from family (whether co-resident or not) was important to the person living with dementia managing well. This is in line with previous research regarding the relationship quality of the person living with dementia and their carer [38]. However, difficulties were encountered both when either the person living with dementia and their carer were now separated, or they had moved in together. This led to marked differences in how child and spousal carers had experienced lockdown, with child carers describing more challenges following changes to living situations affecting both their own and the person living with dementia’s wellbeing and COVID-19 risk. Co-habitation has been noted to increase burden on carers [39] to a greater extent for adult child carers [40]. These residential changes as a result of the COVID-19 pandemic increased the levels of care provision, which is known to be linked to higher levels of psychological distress of both spousal and adult child carers of older adults [41].

Within our sample, when the carer was supporting a parent and was from an ethnic minority, they were often already living with the person living with dementia. This was not the case for the adult child carers of White British background, which could mean that the increased pressure and strain as result of changes to living arrangements are not as applicable to some minority ethnic groups. There are many different cultural interpretations of the nature of caring relationships for people living with dementia [42], which can intersect with, for example, expectations of caring roles, gender roles, stigma or relationships with health and care services [43]. In situations where these intercultural factors led to more established familial care structures, it seemed that families were more able to provide immediate support to the person living with dementia and so avoid additional stress caused by changes to living arrangements. Other than changes to living arrangements, experiences of COVID-19 restrictions and their impacts seemed comparable across participants, regardless of ethnicity.

For people living with dementia and their carers who discussed specific mental health problems, they mostly reported increased anxiety, irritability or apathy in the person living with dementia, in a similar vein to qualitative work conducted in Canada [22]. It may be that these negative experiences were shared more prominently by carers, which is in line with previous research comparing the perspectives of people living with dementia and their carers [44]. However, some carers in our study acknowledged that they might be noticing this more due to their current heightened awareness, either from changes to their living situations or because they were paying more attention and assessing the needs of the person living with dementia more closely.

Quantitative studies conducted with people living with dementia during the COVID-19 pandemic report an increase in similar mental health problems [11–15], and, when comparing this to a survey conducted generally with older people in the UK, similar problems with mental health were reported during the early months of the COVID-19 pandemic [45]. The notable worry for people living with dementia, as mentioned by some of our participants, was whether these problems would remain following the easing of restrictions and if they could recover.

### Strengths and limitations

This study is one of the first to report the perspectives of both people living with dementia and their carers living in the community regarding the impact of initial COVID-19 restrictions in England. The strengths of this study include the sample, which was diverse in regard to ethnicity, gender, carer relationship, accommodation status of the person living with dementia, and dementia diagnoses. Additionally, the research team has extensive experience with qualitative research, and expertise in dementia care, ageing, caring and long-term conditions. However, the limitations include the fact that participants who have difficulty speaking or understanding English or those with difficulty communicating through telephone or video calls are under-represented in our sample. As capacity to consent was an inclusion criterion, and the median date of diagnosis was relatively recent (2019), those in more advanced stages of dementia are also under-represented. Internet or telephone access was required for study participation, and it is likely that our study was not accessible to individuals who may experience heightened digital exclusion, or would not feel comfortable taking part in a remote interview due to, for example, communication problems. This is despite our making accommodations for individuals to take part, including having dyadic interviews with carers, to encourage participation. There is a risk of participation bias in our sample, which may mean that we are missing accounts from individuals who are not coping and finding the current situation more difficult, either due to more severe dementia symptoms or COVID-19 related complications.

### Implications

These findings support the recommendations of having regular routines for people living with dementia [46]. Some of the activities highlighted by participants in this study, such as getting out of the house, either to a garden or a park, meant they had to be physically able, and so more support with mobility aids and alternatives should be considered for those who find this difficult, due to, for example, mobility impairment. Structured routines should also include support for more frequent remote social interactions where this may be lacking due to, for example, technological barriers. Where the mental health of people living with dementia has been impacted negatively by reduced social engagement or less familial support, individuals at heightened risk of negative impacts will need follow up to establish whether they can recover as well as to identify any long-term impact.

Supporting carers of people living with dementia requires particular attention for those who have experienced changes to their caring role, due, for example, to changes in living situations as well as social restrictions affecting carer roles within a larger family. Further research which includes the wider social support circle of a person living with dementia, not just one identified ‘carer’, could increase our understanding of how these wider relationships and networks support people living with dementia. Investigations of these wider networks, especially in crises where caring systems are forced to change, would provide deeper understanding of the impact of such changes during periods of increased social isolation. Future research could also explore in greater depth the impact of the COVID-19 pandemic on carers specifically. Additionally, this study also provides further evidence for the impact of the lack of formal day and respite care [13, 22].

The findings of this study emphasise several opportunities to improve the wellbeing of people living with dementia and their carers. It is important to acknowledge the autonomy of the person living with dementia, and provide them with support in order to make informed decisions within their capacity to make such decisions in situations of increased risk, for example clearly explaining the personal implications of social restrictions enforced to manage COVID-19 risk. For professionals engaging in discussions about risk management, there should be understanding of the communication and conceptualisation of risk in the mind of the person living with dementia and their carer in order to establish effective informed risk-related decision-making [47].

This study also emphasises the ethical dilemmas and difficulties of providing best practice person-centred care during a pandemic, which at times may seem to require antithetical approaches. This subject has been more widely discussed with regard to excluding visitors to care homes to reduce COVID-19 transmission despite the impact on the quality of life of residents with (and without) dementia [18], but is equally applicable to the people living with dementia who live at home. While previous research regarding risk taking and decision making by people living with dementia was conducted before this pandemic [48, 49], similar guidelines should be developed in order to enable people living with dementia to make their own informed decisions or plans where possible.

## Conclusion

COVID-19 restrictions have affected people living with dementia and their carers, and this study has shown the positive influence of familial support and caring relationships on a person living with dementia. Changes to living arrangements between carers and people living with dementia may strain caring relationships due to lack of respite for carers and reduced independence for people living with dementia, although this was largely discussed by White British adult child carers who had not previously lived with the person living with dementia and may have different caring expectations and norms to spousal carers and to those of other ethnic or cultural backgrounds. Adapting previous routines so people could continue living at home was reported to be beneficial, as was being able to get outside the home for a socially distanced walk or gardening. The increased negative psychological symptoms reported by people living with dementia were attributed to reduced social engagement, with a concern about whether these would improve following the easing of restrictions. Future research should continue to explore the impact of social restrictions on people living with dementia, especially those that do not have carers and develop supportive responses.

## Supplementary Information

**Additional file 1.** Topic guides. Interview topic guides for person living with dementia and carer.

## Data Availability

The datasets generated and analysed during the current study are not publicly available due ethical approval regulations limiting the availability of the data.

## Abbreviation

UK: United Kingdom
