# Supplementary material for: Life under lockdown and social restrictions - the experiences of people living with dementia and their carers during the COVID-19 pandemic in England
Source: BMC Geriatr. 2021 May 10;21:301. doi: 10.1186/s12877-021-02257-z (PMC8107803; doi:10.1186/s12877-021-02257-z)
Supplement: Supplementary file 1 — Additional file 1. Topic guides. Interview topic guides for person living with dementia and carer. [file 12877_2021_2257_MOESM1_ESM.pdf]

Additional files for *Life under lockdown and social restrictions - the experiences of people living with dementia and their carers during the COVID-19 pandemic in England*

Interview topic guides for people living with dementia and carers

HEARD Interview topic guide for service users

Introduction, review of consent, confirmation of permission to audio record the interview.

Present the ECOMAP, and work out a rough sketch of the current situation of the support they are receiving. Emphasise this is a draft and as you talk you can fill out or add in.

'What would you usually do on a regular day at the moment?'

'How would you say your health is right now?'

'Do you currently have any health conditions?'

'Who do you talk to about this?'

Current experiences of health care (in context of COVID-19):

Can you tell me a little bit about how you are experiencing your health care at the moment?

Have you been in touch with your health care providers? (e.g. GP/doctor, nurse, pharmacist, memory clinic)

If yes-

- In what ways are you staying connected with them?
- What was this experience (eg telephone instead of face to face) like for you?
- What did you discuss with them (e.g new concerns, ongoing issues)?
- How are you finding discussing things remotely?
- Are there any issues you are coming across?
- What went well about the experience?
- What could be improved? How?

If no-

- Why not/what is holding you back (if applicable)?
- When might you get in touch?
- Who would you contact?
- How would you get in touch if you needed to?
- What might help you access healthcare if you needed to?

SECTION 1 – INTIAL EXPERIENCE WITH POST-DIAGNOSTIC CARE

Could you tell me about your experiences of health care related to your dementia?

During the interview, prompt for:

- Health care services you have been offered and accepted or turned down
- Your original expectations and what you were looking for (was that met?)
- What you like about current services, what you dislike about services
- What you think could be improved, or if anything is missing
- Thinking ahead, do you think you might want different support from healthcare?

SECTION 2 – SPECIFIC DEMENTIA CARE

If the individual is comfortable talking about their dementia care specify as such, otherwise revert to the language they are using to describe their situation.

The following questions will be about what they felt they needed when they first received their diagnosis. Prompt to think back to their first impressions of how things were and what they are like now.

- What was your first impression of the health care services you interacted with after your diagnosis?
- Was there specific help you sought out or were directed to? What was the reason for this?
- Was there something that was offered to you that you did not end up using? What was the reason for this?
- Was there something you feel you did not get help for straight away?
- What would you recommend for someone who has recently received a diagnosis?

SECTION 3 – SPECIFIC SERVICE EXPERIENCE

If previous mention of a specific service, circle back to this and explore more.

The next experiences we are interested in are about how you feel your memory problems were addressed in [x] (your GP practice, nurses, pharmacist, health care assistants, receptionists, e.g.)

- How have you found your [x] after your diagnosis? (prompt also other professionals)
- How has your [x] been involved in any of the care you received for your memory problems? (prompt for other health problems if negative response)
- How would you like your [x] to be involved in your care?

Is there anything else you would like to tell us about the support you have received for your problems with memory and thinking?

Thank the individual, reminder about confidentiality and following interview regarding any changes next contact will be in roughly 3 months.

HEARD Interview topic guide for carers

Introduction, review of consent, confirmation of permission to audio record the interview.

May I start by asking what your relation is to the person you support? Describe the ECOMAP and update/add any additions with a different colour from the carer perspective.

'What would you usually do on a regular day at the moment?'

'How would you say their health is right now?'

'Do they currently have any health conditions?'

'Who do you talk to about this?'

What have you found difficult when providing support for [person with dementia] in the current situation?

What have you found easier when providing support for [person with dementia] in the current situation?

Could you explain anything that has changed with how you or the person you support engage with your health care providers?

- What did you discuss (new concerns vs ongoing issues)?
- How are you engaging with health care providers and how are you finding this?
- How are you finding these changes?

SECTION 1 – INTIAL EXPERIENCE WITH POST-DIAGNOSTIC CARE

Could you tell me about your experiences of health care for the dementia of the person you care for following the diagnosis?

During the interview, prompt for:

- Health care services you have initiated and have accepted or turned down
- Your original expectations and what you were looking for (was that met?)
- What you like about current services, what you dislike about services
- What you think could be improved, or if anything is missing
- Thinking ahead, do you think you might want different support from healthcare?
- Do you see the same GP most of the time? How do you find the usual appointments? Do they ask how you're doing as well?

SECTION 2 – SPECIFIC DEMENTIA CARE

The following questions will be about what they felt was needed after the diagnosis of the person they are caring for. Prompt to think back to their first impressions of how things were and what they are like now.

- What was your first impression of the health care services you interacted with?
- Was there specific help you sought out or were directed to? What was the reason for this?
- Was there something that was offered to you that you did not end up using? What was the reason for this?
- Was there something you feel you did not get help for straight away?

- What would you recommend for someone who has recently received a diagnosis?

SECTION 3 – PRIMARY CARE DEMENTIA EXPERIENCE

If previous mention of a specific service, circle back to this and explore more.

The next experiences we are interested in are about how you feel any issues with memory problems were addressed in [x] (your GP practice, nurses, pharmacist, health care assistants, receptionists, e.g.)

- Do you accompany your relative/friend to regular appointments?
- How have you found your [x] following the diagnosis? (prompt also other professionals)
- How has [x] been involved in any of the care your relative has received for their memory problems? (prompt for other health problems if negative response)
- How would you like [x] to be involved in their care?
- How has this changed since the Covid-19 restrictions?

Is there anything else you would like to tell us about the support your relative/friend has received for problems related to their dementia?

Thank the individual, reminder about confidentiality and following interview regarding any changes first contact will be in roughly 3 months.
